# Mitochondrial ribosomal proteins involved in tellurite resistance in yeast *Saccharomyces cerevisiae*

**DOI:** 10.1038/s41598-018-30479-6

**Published:** 2018-08-13

**Authors:** Paola Pontieri, Hans Hartings, Marco Di Salvo, Domenica R. Massardo, Mario De Stefano, Graziano Pizzolante, Roberta Romano, Jacopo Troisi, Angelica Del Giudice, Pietro Alifano, Luigi Del Giudice

**Affiliations:** 1 Istituto di Bioscienze e BioRisorse-UOS Portici-CNR c/o Dipartimento di Biologia, Sezione di Igiene, Via Mezzocannone 16, Napoli, 80134 Italy; 2 Consiglio per la ricerca in agricoltura e l’analisi dell’economia agraria, Via Stezzano 24, Bergamo, 24126 Italy; 3 Dipartimento di Scienze e Tecnologie Biologiche e Ambientali, Università del Salento, Lecce, 73100 Italy; 4 Dipartimento di Scienze Ambientali, Seconda Università degli Studi di Napoli, Via A. Vivaldi 43, Caserta, 81100 Italy; 5 ZooPlantLab, Dipartimento di Biotecnologie e Bioscienze, Università di Milano-Bicocca, Piazza della Scienza 2, Milano, 20126 Italy; 6 Dipartimento di Ingegneria Civile, Chimica, Ambientale e dei Materiali (DICAM), Università di Bologna, Via Terracini 28, Bologna, 40131 Italy; 7 Theoreo srl - Spin off dell’ Università di Salerno, Via Salvatore Derenzi 50, Montecorvino Pugliano, 84125 (SA) Italy; 8 Amb di allergologia Osp Martini asl città di Torino, via Tofane 71, Torino, 10171 Italy

## Abstract

A considerable body of evidence links together mitochondrial dysfunctions, toxic action of metalloid oxyanions, and system and neurodegenerative disorders. In this study we have used the model yeast *Saccharomyces cerevisiae* to investigate the genetic determinants associated with tellurite resistance/sensitivity. Nitrosoguanidine-induced K_2_TeO_3_-resistant mutants were isolated, and one of these mutants, named Sc57-Te_5_^R^, was characterized. Both random spore analysis and tetrad analysis and growth of heterozygous (Te^S^/Te_5_^R^) diploid from Sc57-Te_5_^R^ mutant revealed that nuclear and recessive mutation(s) was responsible for the resistance. To get insight into the mechanisms responsible for K_2_TeO_3_-resistance, RNA microarray analyses were performed with K_2_TeO_3_-treated and untreated Sc57-Te_5_^R^ cells. A total of 372 differentially expressed loci were identified corresponding to 6.37% of the *S. cerevisiae* transcriptome. Of these, 288 transcripts were up-regulated upon K_2_TeO_3_ treatment. About half of up-regulated transcripts were associated with the following molecular functions: oxidoreductase activity, structural constituent of cell wall, transporter activity. Comparative whole-genome sequencing allowed us to identify nucleotide variants distinguishing Sc57-Te_5_^R^ from parental strain Sc57. We detected 15 CDS-inactivating mutations, and found that 3 of them affected genes coding mitochondrial ribosomal proteins (*MRPL44* and *NAM9*) and mitochondrial ribosomal biogenesis (*GEP3*) pointing out to alteration of mitochondrial ribosome as main determinant of tellurite resistance.

## Introduction

Tellurium (Te) is a highly toxic metalloid belonging to the chalcogen family, chemically related to selenium and sulfur. Te is a relatively rare on Earth’s crust where it is commonly found in combination with many metals forming metal tellurides^1^. Concentrations of Te compounds may be elevated in soils and waters in proximity of wastewater treatment plants of certain industrial settings. These compounds, and in particular potassium tellurite (K_2_TeO_3_), are toxic to most microorganisms^2,3^. It is believed that tellurite toxicity is mainly due to its ability to act as a strong oxidizer toward a variety of cellular components^4,5^. It has been recently argued that tellurite could exert its toxic effect through the generation of reactive oxygen species (ROS). Indeed, intracellular ROS such as hydrogen peroxide (H_2_O_2_), superoxide anion (O_2_^−^) and hydroxyl radical (^•^OH) are common by-products of the aerobic metabolism that are produced upon exposure of cells to free radical-generating molecules like metals and metalloids^6^. Nevertheless, naturally occurring tellurite resistant bacteria may be isolated from polluted environments, and they often reduce toxic tellurite to less toxic elemental form Te^0^ that accumulates inside the cell forming black deposits^1,2,7,8^. On a mechanistic point of view, tellurite enters bacterial cells thorough the phosphate transporter, and then it is reduced by several oxidoreductases such as nitrate reductase^2^ and respiratory chain terminal oxidases^8^, glutathione and other thiol-carrying molecules^9^. Other proposed mechanisms mediating tellurite resistance involve cysteine-metabolizing enzymes and methyl transferases^1–3,10^. Bacterial Te resistance (Te^R^) genetic determinants may be located on either plasmids^1^ or chromosome^1,11,12^, and most of these determinants mediate tellurite resistance by unknown mechanisms.

Not only prokaryotes, but also eukaryotes such as fungi and yeasts, in addition to plant and animal tissues, have the ability to reduce tellurite by various reactions that lead to accumulation of black Te^0^ precipitates^1,13–15^. Interestingly, Te toxicity has been related to the etiopathogenesis of several neurodegenerative disorders^16,17^. In rat Te causes cerebral lipofuscinosis with cognitive impairment, a pathological picture that is similar to that described in Kuf’s disease that has some clinical and neuropathological traits reminiscent of the Alzheimer’s disease^16^. Te may impair mitochondrial functions resulting in defective energy metabolism and increased oxidative damage, which may be relevant to the pathogenesis of neurodegenerative disorders. Moreover, tellurite resistance and/or its underlying mechanisms have been directly/indirectly implicated in Friedreich’s ataxia^18^, an autosomal recessive neurodegenerative disorder that is caused by mutations affecting frataxin gene (*FRDA*) coding for a mitochondrial matrix protein^19^. Although this protein has been implicated in mitochondrial iron homeostasis, its function has not yet been completely elucidated. Frataxin homolog genes were found in *Caenorhabditis elegans* roundworm and *S. cerevisiae* yeast^19^. A frataxin homologue (*cyaY*) is also present in Proteobacteria including purple bacteria, which are believed to be the closest living relatives to mitochondria, suggesting that *FRDA* evolved from *cyaY* from the bacterial ancestor of mitochondria^20^. Multiple sequence alignment of frataxin with homologous proteins from eukaryotes and bacteria showed that the conserved core region between frataxin and CyaY proteins^21^ share sequence homologies with the N-terminal region of small proteins which confer resistance to Te (Te^R^) compounds^2,20^. Although the biochemical function of tellurite resistance proteins is currently unknown, it is a generally accepted hypothesis that Te^R^ results from conversion of tellurite to a less toxic compound. However, since tellurium compounds are very rare in the environment it appears unlikely that primary function of the Te^R^ protein is to detoxify tellurium. On the other hand, the extensive diffusion of Te^R^ proteins among bacteria suggests an important role in cellular life. Hence, the identification of mutations conferring tellurite resistance in yeasts should provide insights into cellular functions of fundamental importance to unveil the molecular bases of certain human diseases.

## Results

### Te^0^ precipitation test

**Figure 1:**
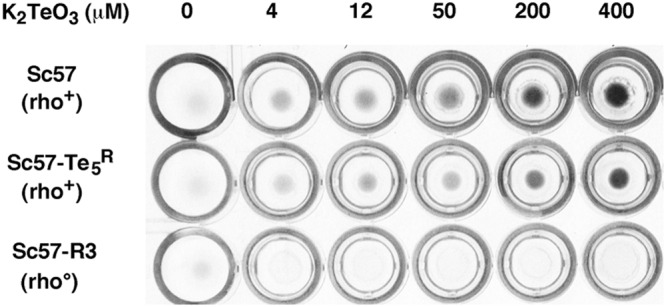
Tellurium precipitation test in microtiter plate. Fresh stationary phase preculture of Sc57, Sc57-Te_5_^R^ or Sc57-R3 yeast strains were inoculated in YED broth plus increasing amounts of K_2_TeO_3_.

Tellurium precipitation (likely Te^0^) was observed in the presence of increasing amounts of K_2_TeO_3_ in microtiter plate assays (Fig. 1). The amount of Te^0^ deposited in microtiter wells by respiratory-competent strain Sc57 increased as a function of K_2_TeO_3_ concentration. Conversely, Te^0^ precipitation could not be detected either when Sc57 was cultivated in YEG medium (data not shown), or when respiratory-deficient Sc57- R3, cultured in YED medium, was used (Fig. 1). The different results of tellurium precipitation assays with rho^+^ and rho^0^ strains, and the different tolerance to K_2_TeO_3_ of rho^+^ yeast cells in YED with respect to YEG suggested that Te was affecting mitochondrial activities, confirming previous work^15^.

### Transmission electron microscopy

**Figure 2:**
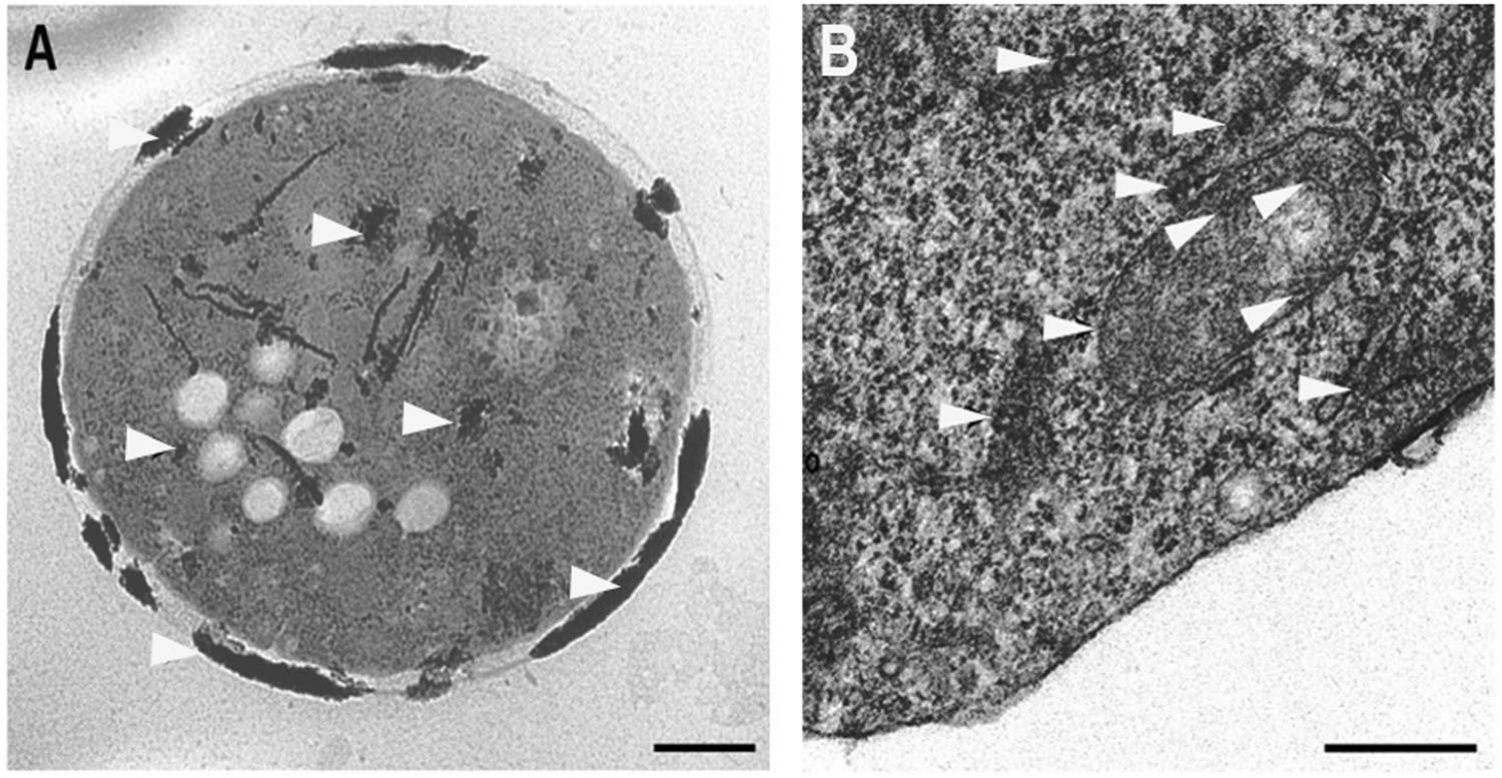
TEM micrograph sections of *S. cerevisiae* Sc57 yeast cells grown in the presence of K_2_TeO_3_. (**A**) Sc57 cell. Scale bar: 2.5 μm. Arrowheads indicate elemental Tellurium (Te^0^) deposits. (**B**) Details of the image in A showing elemental Te^0^ grains tended to form deposits along the cell wall, in the cytoplasmic matrix, in the mitochondrial cristae and in the interspaces between the mitochondrial internal and external membranes (arrowheads). Scale bar: 1 μm.

Images from transmission electron microscopy (TEM) demonstrated that Te^0^ formed large deposits along the cell wall of yeast cells (Fig. 2A, arrowheads), and smaller deposits in the cytoplasmic matrix (Fig. 2B, arrowheads). In yeast cells mitochondria, Te^0^ grains were also detected either on the internal membranes (cristae), or in the interspaces between the internal and external membranes (Fig. 2B, arrowheads). This finding further indicated that mitochondria could play a key role in tellurite toxicity.

### Isolation and physiological characterization of the tellurite resistant mutant

Since *S. cerevisiae* rho^+^ cells were able to grow in glucose medium with increasing concentrations of K_2_TeO_3_ causing blackening of either the culture medium (Fig. 1) or the plated colonies, the isolation of tellurite resistant mutants was possible only in YEG medium containing glycerol as non-fermentable carbon source^15^. Growth of the parental *S. cerevisiae* wild type strain Sc57 is inhibited by 12.5 μg/ml K_2_TeO_3_ in YEG broth, whilst is inhibited by 25 μg/ml K_2_TeO_3_ in YEG plates. The strain Sc57 was mutagenized with NTG (40 μg/ml; 28 °C; 50 min) to yield a surviving fraction of 5–10%. Cells were plated onto glycerol medium (YEG plates) containing 100 μg/ml K_2_TeO_3_. Mutants appeared at a frequency of about 5 per 10^7^ cells after 10 days of incubation at 25 °C. No spontaneous mutants appeared among 10^9^ cells under the same conditions. Altogether 3400 colonies were screened by replica plating for resistance to K_2_TeO_3_. Nine out 3400 colonies examined showed the Te^R^ phenotype. These putative mutants were purified three times by isolation and only four of them presented a stable Te^R^ phenotype. One of these mutants, named Sc57-Te_5_^R^ (rho^+^), was subsequently characterized.

**Figure 3:**
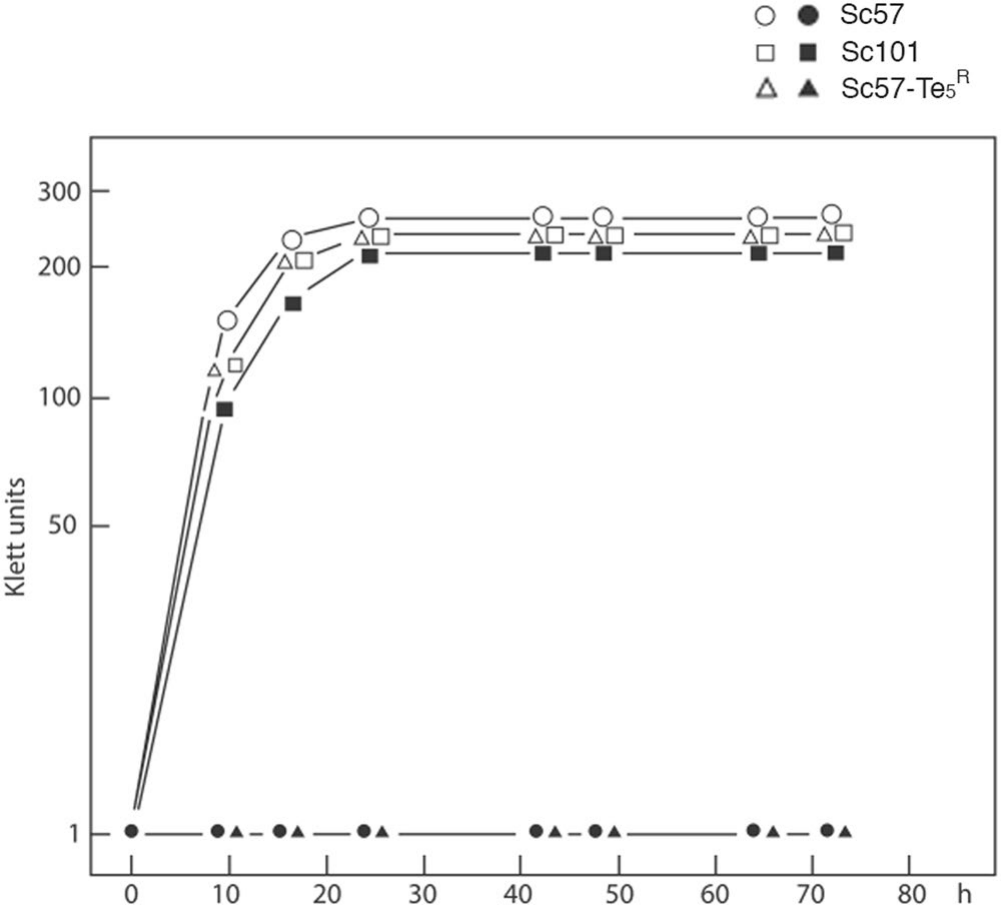
Growth curves of *S. cerevisiae* strains in non-fermentable media (YEG) in presence (closed symbol) or absence (open symbol) of 100 *µg/ml* K_2_TeO_3_: ◯ ● Sc 57. △ ▲ Sc 101. □ ■ Sc 57-Te5R.

Growth curves of the parental strain Sc57 Te^S^ as well as the mutant Sc57-Te_5_^R^ in YEG broth in presence and absence of 100 µg/ml K-Te are presented in Fig. 3. As shown, growth of the parental strain Sc57 Te^S^ was completely inhibited, whereas a growth kinetic similar to that of strains in K_2_TeO_3_ free medium was observed for the mutant Sc57-Te_5_^R^. Besides, it was also performed the growth by dropping for controlling the resistance to K_2_TeO_3_ on solid medium with glycerol (YEG) as shown in Supplementary Fig. S1. It is well evident in Supplementary Fig. S1 the colony growth of both the mutant Sc57-Te_5_^R^ and the parental Sc57 Te^S^ strains without tellurite, while with tellurite only the mutant Sc57-Te_5_^R^ was able to grow.

No particular difference were observed by TEM in tellurium deposit along the cell wall and in the cytoplasmic matrix between Sc57 and Sc57-Te5R yeast cells grown in YED whereas a slight reduction of Te0 deposits seemed to be evident in the mitochondria of Sc57-Te5R (data not shown).

### Genetic analysis of Sc57-Te5R

The mutant Sc57-Te_5_^R^ was crossed with strain Sc101 (Te^S^) and diploids selected on minimal medium (YNG supplemented with appropriate nutritionals). Approximately 2,000 individual diploid clones were assayed by replica plating on K_2_TeO_3_ containing medium. All of them proved to be sensitive to the drug, indicating a recessive nuclear mutation. Recessive diploids from the cross were sporulated and genetic analysis was carried out either by random spore analysis or by tetrad analysis. With reference to random spore analysis, 2,300 spores were analyzed showing a nearly 1:1 segregation of auxotrophic markers, while 47% of the progeny was wild type tellurite sensitive and 53% showed a tellurite resistant phenotype. These data demonstrate that the tellurite resistant phenotype is associated with nuclear mutation(s). The nuclear origin of resistance to tellurite is being confirmed by 12 complete tetrads analyzed. In all cases the tellurite sensitive versus resistant phenotype segregated 2 Te^S^: 2 Te^R^. In all tetrads the nuclear auxotrophic markers segregate 2:2.

### Cloning and homology analysis of PCR frataxin sequences

The homology between frataxin family to bacterial protein family that are associated to resistance to tellurium, prompted us to look at possible mutations in the yeast frataxin gene as responsible for Te^R^ phenotype. We thus amplified, cloned in *E. coli*, and sequenced the frataxin gene from Sc57 and Sc57-Te_5_^R^. A total of 10 clones were examined from each yeast strain. The alignment of sequences highlighted the almost total correspondence between all the sequences obtained and those deposited in the database, and the absence of differences between Sc57 and Sc57-Te_5_^R^ frataxin genes. Therefore, the phenotypic differences observed between Sc57 (Te^S^) and Sc57-Te_5_^R^ could not be attributed to the nucleotide variants in the frataxin locus.

### Gene expression measurement by RNA microarray

In order to measure fluctuations in gene expression, Affymetrix genechips (GeneChip Yeast Genome 2.0 Array) were utilized, providing a comprehensive coverage of both *S. cerevisiae* (5,841 probe sets) and *S. pombe* (5,021 probe sets). Total RNA, obtained from Sc57-Te_5_^R^ strain treated with K_2_TeO_3_ and from an untreated control was used to prepare molecular probes and hybridize the yeast microarray. The dataset obtained was analyzed using the FlexArray 1.6.1 software suite (Génome Québec and McGill University). First, raw data were normalized using the PLIER (Probe Logarithmic Intensity Error) algorithm. Subsequently, significant expression differences were identified by means of t-tests. Only differently expressed sequences with p-values < 0.01 and expression ratios > 2.0 between induced and non-induced yeast cells were taken into consideration. In this manner, a total of 370 differentially expressed loci were identified, corresponding to 6.37% of the *S. cerevisiae* transcriptome. Of these, 286 sequences appeared up-regulated upon tellurite induction (Supplementary Table S1), while 84 sequences resulted down-regulated (Supplementary Table S2). Differentially expressed sequences were analyzed with «The Database for Annotation, Visualization and Integrated Discovery (DAVID) v6.8» (https://david.ncifcrf.gov/), to identify enriched groups of genes with altered expression values associated with the applied treatment. Down-regulated sequences did not, but marginally, combine into larger groups after gene enrichment by molecular function (65% of the sequences did not combine into groups). Two groups with a reasonable number of genes could be identified. A first group, counting 15 sequences, was related with «transferase activity» (GO:0016740), while a second group of 10 sequences was related with «oxidoreductase activity» (GO: 0016491). Up-regulated sequences, on the other hand, combined into several groups with distinct molecular functions. Sequences aggregated into 39 groups after GO enrichment analysis for «Biological Processes», while 17 enriched groups were obtained for the «Molecular Function» sub-ontology group (Table 1). Taking into consideration the «Cellular Component» sub-ontology group, a large cluster containing 101 (35.4%) of the differentially expressed sequences could be identified. These sequences were all correlated with GO:0031224 (intrinsic component of membrane). A smaller group of 26 sequences (9.1%), correlating with GO:0016022 (endoplasmic reticulum) could also be identified (data not shown).

**Table 1:** Summary of microarray analysis. GO IDs associated with significantly altered gene expression (p < 0.01) and their corresponding terms are listed.

	Term	GO	%	P-Value
BP	transport	GO:0006810	16.35	4.80E-04
	transmembrane transport	GO:0055085	8.34	2.00E-06
	oxidation-reduction process	GO:0055114	7.77	5.60E-03
	ribosome biogenesis	GO:0042254	5.48	3.00E-03
	rRNA processing	GO:0006364	4.91	2.00E-02
	fungal-type cell wall organization	GO:0031505	4.33	3.50E-03
	ion transport	GO:0006811	4.01	2.20E-03
	iron ion homeostasis	GO:0055072	3.43	1.80E-08
	response to stress	GO:0006950	3.19	2.00E-05
	maturation of SSU-rRNA from tricistronic rRNA transcript	GO:0000462	3.19	1.10E-03
	rRNA methylation	GO:0031167	2.62	4.20E-03
	cellular amino acid biosynthetic process	GO:0008652	2.62	6.60E-02
	cellular iron ion homeostasis	GO:0006879	2.29	2.20E-03
	steroid metabolic process	GO:0008202	2.04	3.90E-04
	sterol biosynthetic process	GO:0016126	2.04	9.80E-04
	pseudohyphal growth	GO:0007124	2.04	3.70E-02
	steroid biosynthetic process	GO:0006694	1.72	4.30E-03
	ribosomal large subunit biogenesis	GO:0042273	1.72	8.50E-02
	siderophore transport	GO:0015891	1.47	2.30E-04
	copper ion import	GO:0015677	1.47	9.80E-04
	ergosterol biosynthetic process	GO:0006696	1.47	2.00E-02
	glucose import	GO:0046323	1.47	4.20E-02
	ribosomal small subunit biogenesis	GO:0042274	1.47	4.20E-02
	carbohydrate transport	GO:0008643	1.47	5.70E-02
	copper ion transport	GO:0006825	1.14	1.40E-02
	response to unfolded protein	GO:0006986	1.14	2.20E-02
	sterol transport	GO:0015918	1.14	2.20E-02
	fructose transport	GO:0015755	1.14	3.20E-02
	mannose transport	GO:0015761	1.14	3.20E-02
	siderophore transmembrane transport	GO:0044718	0.90	1.80E-02
	methionine metabolic process	GO:0006555	0.90	3.60E-02
	zinc II ion transport	GO:0006829	0.90	5.80E-02
	iron ion transport	GO:0006826	0.90	8.30E-02
	zinc II ion transmembrane transport	GO:0071577	0.90	8.30E-02
	methionine import	GO:0044690	0.57	8.70E-02
	methionine transport	GO:0015821	0.57	8.70E-02
	peptidyl-lysine modification to peptidyl-hypusine	GO:0008612	0.57	8.70E-02
	urea catabolic process	GO:0043419	0.57	8.70E-02
	arsenate ion transmembrane transport	GO:1901684	0.57	8.70E-02
MF	oxidoreductase activity	GO:0016491	27.33	2.30E-03
	structural constituent of cell wall	GO:0005199	10.87	1.20E-04
	transporter activity	GO:0005215	9.94	3.20E-02
	transcription factor activity	GO:0003700	8.70	3.00E-02
	hydrolase activity. acting on glycosyl bonds	GO:0016798	6.52	5.20E-02
	mannose transmembrane transporter activity	GO:0015578	4.35	2.60E-02
	fructose transmembrane transporter activity	GO:0005353	4.35	2.60E-02
	cyclin-dependent protein serine/threonine kinase regulator activity	GO:0016538	4.35	8.80E-02
	ferric-chelate reductase (NADPH) activity	GO:0052851	3.42	3.60E-02
	iron ion transmembrane transporter activity	GO:0005381	3.42	4.60E-02
	ferric-chelate reductase activity	GO:0000293	3.42	5.80E-02
	monooxygenase activity	GO:0004497	3.42	5.80E-02
	zinc ion transmembrane transporter activity	GO:0005385	3.42	8.30E-02
	glycerol-1-phosphatase activity	GO:0000121	2.17	8.70E-02
	methionine adenosyltransferase activity	GO:0004478	2.17	8.70E-02
	sugar-phosphatase activity	GO:0050308	2.17	8.70E-02

BP – biological process, MF – molecular function, % - percentage of gene sequences included within each group with respect to the total number of sequences identified.

Mitochondrial genes were not differently expressed. However, when the expression of nuclear genes coding for 901 “high-confidence” mitochondrial proteins^22^ was examined, 20 and 17 sequences appeared, respectively, up- and down-regulated in response to K_2_TeO_3_ treatment (Supplementary Tables S1, S2, and Table 2). Among the up-regulated genes there were some involved in carbon and energy metabolism (*HXK2*, *ALD5*, *CYC1*), leucine biosynthesis (*LEU9*), mitochondrial iron homeostasis (*FTR1*, *MMT1, GGC1*), oxidation-reduction processes (*OYE2*), repair of oxidative damage to mitochondrial DNA (*OGG1*), and mitochondrial inner membrane protein assembly (*TIM12*). Among the down-regulated genes we found those involved in 2-methylcitrate pathway (*CIT3, PDH1, ICL2*), glyoxalase system (*GLO4*), redox homeostasis (*AAT1, TRR2*), iron-sulfur cluters assembly (*ISU1*), lipid metabolism (*YAT1, YMR210W, TES1*), and cytochrome oxidase assembly (*COX23*).

**Table 2:** Genes coding for mitochondrial proteins, which were up- or down-regulated upon K_2_TeO_3_ treatment in SC57-Te_5_^R^ strain.

	GeneID	Symbol	Chromosome	Description
Up-regulated	852540	YBR238C	II	Mitochondrial membrane protein with similarity to Rmd9p; not required for respiratory growth but causes a synthetic respiratory defect in combination with rmd9 mutations; transcriptionally up-regulated by TOR; deletion increases life span
	852639	HXK2	VII	Hexokinase isoenzyme 2 that catalyzes phosphorylation of glucose in the cytosol
	852707	SUA5	VII	Single-stranded telomeric DNA-binding protein, required for normal telomere length
	856445	FSH1	VIII	Fsh1p, serine-type hydrolase
	852817	PUS2	VII	Mitochondrial tRNA:pseudouridine synthase
	856804	ALD5	V	Ald5p, acetaldehyde dehydrogenase involved in the biosynthesis of acetate during anaerobic growth on glucose
	854942	OGG1	XIII	Mitochondrial glycosylase/lyase that specifically excises 7,8-dihydro-8-oxoguanine residues located opposite cytosine or thymine residues in DNA, repairs oxidative damage to mitochondrial DNA, contributes to UVA resistance
	853507	CYC1	X	Cyc1p, iso-1-cytochrome c
	852388	TIM12	II	Essential protein of the inner mitochondrial membrane, peripherally localized; component of the TIM22 complex, which is a twin-pore translocase that mediates insertion of numerous multispanning inner membrane proteins
	850636	SSA2	XII	ATP binding protein involved in protein folding and vacuolar import of proteins; member of heat shock protein 70 (HSP70) family
	852401	YMC2	II	Mitochondrial protein, putative inner membrane transporter with a role in oleate metabolism and glutamate biosynthesis; member of the mitochondrial carrier (MCF) family
	851561	MCD1	IV	Essential subunit of the cohesin complex required for sister chromatid cohesion in mitosis and meiosis; apoptosis induces cleavage and translocation of a C-terminal fragment to mitochondria; expression peaks in S phase
	856584	OYE2	VIII	Oye2p, NADPH oxidoreductase; involved in oxidative stress response
	856888	FTR1	V	Ftr1p, ferric iron permease
	855215	MMT1	XIII	Mmt1p; involved in mitochondrial iron export
	852060	UTP6	IV	Nucleolar protein, component of the small subunit (SSU) processome containing the U3 snoRNA that is involved in processing of pre-18S rRNA
	856671	UTR2	V	Chitin transglycosylase that functions in the transfer of chitin to beta(1–6) and beta(1–3) glucans in the cell wall
	851329	GGC1	IV	Ggc1p, mitochondrial GTP/GDP carrier; has a role in mitochondrial iron transport
	1466465	YJL133C-A	X	Putative protein of unknown function; the authentic, non-tagged protein is detected in highly purified mitochondria in high-throughput studies
	854275	LEU9	XV	Alpha-isopropylmalate synthase II (2-isopropylmalate synthase), catalyzes the first step in the leucine biosynthesis pathway
Down-regulated	856108	PDH1	XVI	Mitochondrial protein that participates in respiration, induced by diauxic shift; homologous to *E. coli* PrpD, may take part in the conversion of 2-methylcitrate to 2-methylisocitrate
	850844	PCD1	XII	Pcd1p, Nudix hydrolase for oxidized purine nucleoside triphosphates
	856114	ICL2	XVI	2-methylisocitrate lyase of the mitochondrial matrix, functions in the methylcitrate cycle to catalyze the conversion of 2-methylisocitrate to succinate and pyruvate; ICL2 transcription is repressed by glucose and induced by ethanol
	856107	CIT3	XVI	Cit3p, citrate/2-methylcitrate synthase
	854205	GLO4	XV	Glo4p, mitochondrial glyoxalase II
	852179	BNA4	II	Bna4p, kynurenine 3-monooxygenase; involved in tryptophan degradation; involved in Huntington disease in humans
	852663	MIG2	VII	Mig2p, transcriptional regulator mediating glucose repression
	853145	BNS1	VII	Bns1p; component of FEAR (CDC14 early anaphase) network
	853755	AAT1	XI	Aat1p, mitochondrial aspartate aminotransferase; contributes to the respiratory deficit of yeast frataxin-deficient cells
	856506	TRR2	VIII	Mitochondrial thioredoxin reductase involved in protection against oxidative stress, required with Glr1p to maintain the redox state of Trx3p
	855968	ISU1	XVI	Conserved protein of the mitochondrial matrix, performs a scaffolding function during assembly of iron-sulfur clusters, interacts physically and functionally with yeast frataxin (Yfh1p)
	853477	TES1	X	Peroxisomal acyl-CoA thioesterase likely to be involved in fatty acid oxidation rather than fatty acid synthesis
	855250	YMR210W	XIII	Putative acyltransferase with similarity to Eeb1p and Eht1p, has a minor role in medium-chain fatty acid ethyl ester biosynthesis; may be involved in lipid metabolism and detoxification
	851285	YAT1	I	Outer mitochondrial carnitine acetyltransferase
	856516	COX23	VIII	Cox23p; required for cytochrome oxidase assembly
	855045	RSF1	XIII	Protein required for respiratory growth; localized to both the nucleus and mitochondrion; may interact with transcription factors to mediate the transition to respiratory growth and activate transcription of nuclear and mitochondrial genes
	850861	YLR164W	XII	Mitochondrial inner membrane of unknown function; similar to Tim18p and Sdh4p; expression induced by nitrogen limitation in a GLN3, GAT1-dependent manner

### Comparative genomics of Sc57 and Sc57-Te5^R^ strains

We sought to identify variants introduced in Sc57-Te_5_^R^ strain by the mutate-and-screen method that determined the tellurite resistance phenotype. We thus sequenced the whole genome of Sc57-Te_5_^R^ and parental strain Sc57, and compared them with those of reference strain S288C^23^. We then identified nucleotide variants distinguishing Sc57-Te_5_^R^ from parental strain Sc57. Apart from synonymous substitutions and variants occurring in intergenic regions, we counted a total of 270 variants affecting 217 coding sequences (CDS) in Sc57-Te_5_^R^ (Supplementary Table S3). Among these, 248 were missense mutations, 11 nonsense mutations, 5 sense mutations, 1 frame-shift mutation, and 5 in frame insertions/deletions. Among missense mutations, 128 are conservative and 120 non-conservative based on BLOSUM62 algorithm^24^. All mutations map on nuclear genes consistently with tetrad analysis. We focused on 15 CDS-inactivating mutations (Table 3) and noticed that 3 of them affect single-copy genes coding mitochondrial ribosomal proteins (*MRPL44* and *NAM9*) or a protein that is involved in mitochondrial ribosomal biogenesis (*GEP3*). The other mutations affect gene encoding ribosomal protein (*RPL33A*), histone H2A (*HTA1*), chromatin-silencing protein SIR3 (*SIR3*), phosphoglucomutase (*PGM2*), phosphatidylinositol-3-/phosphoinositide 5-phosphatase (*INP53*), ferric-chelate reductase (*FRE8*), transcriptional regulatory proteins (*CYC8*, *TBS1*), *SEM1* (proteasome regulatory particle lid subunit), *INP1* (a protein involved in peroxisome retention), *CDC40* (pre-mRNA splicing and cell cycle progression factor), and retroviral element (*YHR214C-B*).

**Table 3:** Comparative genomics: summary of CDS-inactivating mutations in SC57-Te_5_^R^.

Chromosome	GeneID	Symbol	Description	CDS length	Amino acid variation
II	852410	*CYC8*	transcription regulator CYC8	967	+TTG 1211/1212
II	852447	*TBS1*	Tbs1p	1095	−G 3233
IV	851811	*HTA1*	histone H2A	133	+G 117/118
IV	851967	*SEM1*	proteasome regulatory particle lid subunit SEM1	90	+A 61/62
IV	851968	*CDC40*	Cdc40p	456	E94*
VIII	856623	*YHR214C-B*	gag-pol fusion protein	1794	E1782*
XII	850736	*FRE8*	putative ferric-chelate reductase	687	C15*
XII	851163	*SIR3*	chromatin-silencing protein SIR3	979	−C 2020
XIII	855131	*PGM2*	phosphoglucomutase PGM2	570	W405*
XIII	855244	*INP1*	Inp1p	421	+T 1085
XIII	855265	*MRPL44*	mitochondrial 54S ribosomal protein YmL44	148	Q5*
XIV	855585	*NAM9*	mitochondrial 37S ribosomal protein NAM9	487	W396*
XV	854276	*INP53*	phosphatidylinositol-3-/phosphoinositide 5-phosphatase INP53	1108	K356*
XV	854380	*GEP3*	Gep3p – Protein required for mitochondrial small subunit biogenesis	557	V373G; K376*
XVI	855960	*RPL33A*	ribosomal 60S subunit protein L33A	283	W25*

### Tellurite resistance in MRPL44, NAM9 and GEP3 knock-out mutants

To evaluate whether the tellurite resistance phenotype could be associated with the 3 CDS-inactivating mutations affecting the mitochondrial ribosomal proteins or the mitochondrial ribosomal biogenesis, *MRPL44, NAM9* and *GEP3* knock-out mutants were tested. All three mutants were available as heterozygous diploid strains derived from BY4743. Two of them (*MRPL44* and *GEP3*) were also available as *MATa* haploid strains derived from BY4741 (Table 4). The results of MIC experiments demonstrated that the two parental strains BY4743 and BY4741 were inhibited by K_2_TeO_3_ concentrations equal or higher than 25 μg/ml in YEG broth (Supplementary Fig. S2). In contrast all three heterozygous diploid derivative *MRPL44, NAM9* and *GEP3* knock-out mutants, and the haploid *MRPL44* knock-out mutant were able to grow at this concentration (Supplementary Fig. S2), and were inhibited at K_2_TeO_3_ concentrations equal or higher than 100 μg/ml (data not shown). The *GEP3* haploid failed to grow in glycerol medium YEG exhibiting a rho^-^ phenotype.

**Table 4:** List of yeast strains, nuclear and mitochondrial genotypes and origins.

Strain	Nuclear genotype	Mitochondrial genotype	Origin
Sc57 = YM654	*a ura3–52 his 3-Δ200 ade2–101 lys2–801 tyr1–501 Te* ^*S*^	rho^+^	M. Johnston
Sc57-Te_5_^R^	*a ura3–52 his 3-Δ200 ade2–101 lys2–801 tyr1–501 Te* ^*R*^	rho^+^	This work
Sc57-R3	*a ura3–52 his 3-Δ200 ade2–101 lys2–801 tyr1–501*	rho^0^	L. Del Giudice
Sc101 = DG1141	*α his3 ∆200 ura3–167 Ty1his3 AI-242 Gal* ^+^ *Te* ^*S*^ *ts*	rho^+^	M. J. Curcio
BY4741	*a his3Δ1 leu2Δ0 met15Δ0 ura3Δ0*	rho^+^	Dharmacon^TM^
BY4742	*α his3Δ1 leu2Δ0 lys2Δ0 ura3Δ0*	rho^+^	Dharmacon^TM^
BY4743	BY4741/BY4742	rho^+^	Dharmacon^TM^
BY4743–20811	BY4743 *mrpl44*::*kanMX*	rho^+^	Dharmacon^TM^
BY4743–27248	BY4743 *nam9:kanMX*	rho^+^	Dharmacon^TM^
BY4743–22461	BY4743 *gep3:kanMX*	rho^+^	Dharmacon^TM^
BY4741–811	BY4741 *mrpl44*::*kanMX*	rho^+^	Dharmacon^TM^
BY4741–2461	BY4741 *gep3:kanMX*	rho^−^	Dharmacon^TM^

## Discussion

The budding yeast has established as a formidable experimental model for the study of the fundamental cell mechanisms and their dysfunctions. In this study the model yeast has been used to investigate the molecular mechanisms underlying tellurite resistance/toxicity, a phenotype that has been associated to several neurodegenerative disorders in humans^16–18]^. We have isolated and characterized at both the genetic and molecular levels an induced mutant of *S. cerevisiae* yeast, resistant to tellurite by means of nitrosoguanidine treatment. The results described here present as a first experiment the evidence of the deposit of elemental tellurium Te^0^ during the growth of the parental strain Sc57 (Te^S^) in YED broth, with glucose as a fermentable carbon source. As demonstrated in Fig. 1, in the growth wells is observed a deposit of elemental tellurium, which increases with increasing of the K_2_TeO_3_ dose. Slightly less deposit is observed with mutant Sc57-Te_5_^R^ strain. The place where elemental tellurium is deposited within the yeast cell was analyzed by TEM. The images show that Te^0^ formed large deposits along the yeast cell wall (Fig. 2A), and smaller deposits in the cytoplasm and mitochondria (inner membrane cristae and interspace between inner and outer membranes) (Fig. 2B, arrows). With reference to the isolation of the mutant strain named Sc57-Te_5_^R^, resistant to 100 μg K_2_TeO_3_ in glycerol medium YEG, it was isolated from the parental strain Sc57 Te^S^ after NTG treatment and successively characterized both genetically and molecularly.

The tellurite resistance phenotype of the mutant strain Sc57-Te_5_^R^ was shown by monitoring the growth of the yeast strains in YEG broth, glycerol complete medium, with and without addition of 100 μg K_2_TeO_3_. In fact, as shown in Fig. 3, growth of the parental strain Sc 57 Te^S^ was completely inhibited in presence of 100 μg K_2_TeO_3_, whereas growth kinetics similar to that of strains in tellurite free medium was observed for the mutant Sc57-Te_5_^R^ strain. Besides, the tellurite resistance phenotype was also controlled by dropping the yeast cultures on YEG plates, glycerol complete solid medium, with and without addition of 100 μg K_2_TeO_3_, respectively. As shown in Supplementary Fig. S1, both colonies of Sc 57 Te^S^ strain and Sc57-Te_5_^R^ are growing on YEG without addition of tellurite, while on YEG plate with 100 μg K_2_TeO_3_ is observed only growth of the mutant strain Sc57-Te_5_^R^.

To investigate the molecular mechanisms involved in K_2_TeO_3_ resistance/toxicity, the complete transcript profile of Sc57-Te_5_^R^ cells treated with tellurite and from an untreated Sc57-Te_5_^R^ control was analyzed by RNA microarray. A total of 372 differentially expressed loci could be identified, corresponding to 6.37% of the *S. cerevisiae* transcriptome. Of these, 288 sequences appeared up-regulated upon K_2_TeO_3_ treatment (Supplementary Table S1), while 84 sequences resulted down-regulated (Supplementary Table S2). As previously mentioned, Te^0^ deposition in Sc57-Te_5_^R^ cells, was found predominantly along the cell wall, in the cytoplasmic matrix, and the internal membrane of mitochondrial cristae (Fig. 2). This finding is in good agreement with the obtained expression patterns showing an increased expression of a large group (16.35%) of sequences involved in membrane transport (GO:0006810; GO0055085; GO:0005199; GO:0005215 in Table 1).

Curiously, while tellurium deposition in bacteria is commonly associated with Te(IV) resistance^2^ and in yeast, conversely, with Te(IV) susceptibility^25^, the Sc57-Te_5_^R^ strain exhibits a resistant phenotype in spite of an evident Te^0^ accumulation. A common response of *S. cerevisiae* to metal exposure regards the suppression of genes involved in ribosomal biogenesis and translation^26–29^. Again, the Sc57-Te_5_^R^ strain exhibited an opposite response pattern, since several ribosome biogenesis related sequences exhibit an augmented expression upon tellurite induction (GO:0042254; GO:0042273; GO:0042274 in Table 1), an indication of the resistant phenotype of this strain. Finally, the Sc57-Te_5_^R^ strain exhibited an enhanced expression of sequences involved in general defense functions such as (*i*) a general stress response (GO:0006950 in Table 1), (*ii*) metal-specific detoxification strategies such as the chelation of metal ions via siderophores (GO:0015891; GO:0044718 in Table 1), (*iii*) the sugar/carbohydrate metabolism (GO:0046323; GO:0015755; GO:0015761 in Table 1); and (*iv*) metal-ion homeostasis and transport (GO:006811; GO:0055072; GO:0006879; GO:0015677; GO:0006825; GO:0006829; GO:0006826; GO:0071577 in Table 1) as expected during growth under metal exposure^27,30^.

The expression of yeast frataxin gene (*YFH1*) was not significantly affected. However, when expression of nuclear genes coding for 901 “high-confidence” mitochondrial proteins^22^ was analyzed, two sequences dealing with frataxin gene function were found to be down-regulated upon K_2_TeO_3_ treatment: *ISU1* coding for a protein of the mitochondrial matrix, which plays scaffolding function during iron-sulfur clusters assembly, and physically and functionally interacts with yeast frataxin^31–33^, and *AAT1* coding for mitochondrial aspartate aminotransferase, which contributes to the respiratory deficit of yeast frataxin-deficient cells^34^ (Table 2). *ISU1* down-regulation might represent a defensive response toward tellurite-induced disabling of iron-sulfur clusters that leads to release ferrous ions^35^, which in the presence of hydrogen peroxide may generate DNA-damaging hydroxyl radicals^36^. Iron-sulfur clusters disabling may also explain the observed alterations of the expression of genes involved in mitochondrial carbon and energy metabolism, iron homeostasis, redox balance, and oxidative stress response (Table 2). This view is also consistent with the observed down-regulation of *AAT1*. Indeed, decreased activity of the mitochondrial aspartate aminotransferase, which functions in tandem with malate dehydrogenase in the malate/aspartate NADH shuttle, was observed in yeasts lacking mitochondrial DNA or mutants deficients in iron-sulfur cluster assembly including frataxin-deficient cells^34^. Down-regulation of the three key genes of the 2-methylcitrate pathway (*CIT3, PDH1, ICL2*), which is devoted to utilization of propionyl-CoA that is derived by beta-oxidation of odd-chain fatty acids, degradation of amino acids including isoleucine, methionine and valine is well placed in this context. Indeed, *PDH1* encodes a 2-methylcitrate dehydratase that is homolog to PrpD of *E. coli*, a monomeric iron-sulfur protein with an unstable [2Fe-2S] cluster^37^.

Both tetrad analysis and random spore analysis confirmed the K_2_TeO_3_ resistance phenotype of the mutant strain Sc57-Te_5_^R^ to be both a recessive and chromosomally inherited character, not associated with nucleotide variants in the frataxin locus. The results of comparative whole-genome shotgun sequencing allowed us to identify numerous nucleotide variants distinguishing Sc57-Te_5_^R^ from parental strain Sc57. Indeed, apart from synonymous substitutions and variants occurring in intergenic regions, we counted a total of 270 variants affecting 217 coding sequences (CDS) in Sc57-Te_5_^R^ (Supplementary Table S3). Among these, 248 were missense mutations (of which 128 conservative and 120 non-conservative), 11 nonsense mutations, 5 sense mutations, 1 frame-shift mutation, and 5 in frame insertions/deletions. Such a high number of variants were probably due the mutagenic treatment with the potent NTG, and possibly to non-conservative mutation affecting the DNA mismatch-repair gene MSH4 (Supplementary Table S3), which may have generated a mutator phenotype during strain selection. In Sc57-Te_5_^R^ we identified 15 CDS-inactivating mutations. Some of them affect essential genes that are represented in more than one copy on the yeast genome. Three of them, however, inactivate single-copy genes coding mitochondrial ribosomal proteins (*MRPL44* and *NAM9*) or a protein that is involved in mitochondrial ribosomal biogenesis (*GEP3*). This result points out to alteration of mitochondrial ribosome as a main determinant of tellurite resistance consistently with previous findings^15^. This finding, which was supported by measurement of tellurite resistance in *MRPL44, NAM9* and *GEP3* single knock-out mutants (Supplementary Fig. S2), is of great interest also because mitochondrial ribosomal protein genes are candidate genes for many systemic and neurodegenerative diseases in humans including spinocerebellar ataxia with blindness and deafness syndrome (SCABD), autosomal dominant and recessive nonsyndromic sensorineural disease (DFNA and DFNB), multiple mitochondrial dysfunctions (MMDFS), vacuolar neuromyopathy, Moebius syndrome, Leigh syndrome, Usher syndrome, Di George syndrome, Joubert syndrome, Russell-Silver syndrome, Alzheimer disease, Parkinson disease, dilated cardiomyopathy 1E, cataract and diabetes^38^.

In particular, *MRPL44* whose CDS is early interrupted by Q5* nonsense Sc57-Te_5_^R^ (Table 3) encodes a mitochondrial 54S ribosomal protein that is thought to be located in close proximity to the tunnel exit of yeast mitochondrial ribosome^39^. In human mitochondria MRPL44 protein seems to be involved in the assembly/stability of nascent mitochondrial polypeptides exiting the ribosome^40^. A role of this protein in regulating the expression of mtDNA-encoded genes has been also postulated^41^. Reduced levels of this protein mildly affect de novo mitochondrial translation, but lead to large ribosome assembly defect and complex IV deficiency^40^. MRPL44 mutations cause a slowly progressive multisystem disease with childhood-onset hypertrophic cardiomyopathy and neurological and neuro-ophthalmological impairment that occurs during the second and third decades of life^42^.

*NAM9* (also known as *MNA6*) encodes mitochondrial 37S ribosomal protein that is related to the S4 small subunit ribosomal protein of bacteria, which plays a key role in ribosome assembly and translational fidelity^43^. However, at variance with the bacterial homologues, Nam9p contains an additional C-terminal extension (CTE) that shows no obvious homology with any known protein. In Sc57-Te_5_^R^ W396* nonsense prematurely interrupts the CTE (Table 3). Disruption of *NAM9* perturbs mitochondrial DNA integrity and causes respiratory deficiency^44,45^, while amino acid substitution in the C-terminal domain causes temperature-dependent loss of the 15S rRNA^44^. *GEP3* (also known as *MTG3*) is an inner membrane mitochondrial protein that is required for mitochondrial ribosome small subunit biogenesis^46^. Null mutant is defective in respiration and in maturation of 15S rRNA^46^. In Sc57-Te_5_^R^ K376* nonsense interrupts the CDS of *GEP3*.

In conclusion, in Sc57-Te_5_^R^ alteration of mitochondrial ribosome seem to be responsible for tellurite resistance. Based on literature data, common traits of all these mutations are assembly defect, perturbation of mitochondrial DNA integrity and partial respiratory deficiency suggesting that toxicity of tellurite is mainly associated with its reduction into Te^0^ by respiratory chain of yeast mitochondria. Indeed, this process may generate electron leakage leading to release of ROS and induction of cellular oxidative stress. This conclusion is consistent with both RNA microarray data with K_2_TeO_3_ exposed and control yeast cells (Table 1, Supplementary Tables S1 and S2) and C15* nonsense that inactivate the *FRE8* gene coding for a NADPH oxidase. It has been recently demonstrated that FRE8 in *Candida albicans* produces a burst of ROS during fungal morphogenesis^47^, and, thus, *FRE8* mutation may thus further contribute to tellurite resistance phenotype in Sc57-Te_5_^R^ strain by further lowering ROS levels in K_2_TeO_3_ exposed cells. Altogether our findings may help to understand in the yeast model the complex relationships between the mitochondrial biogenesis and functionality, the toxic action of metalloid oxyanions such as tellurite, and the cellular induction and response to ROS, thereby shedding light on human pathologies that involve these mechanisms.

## Materials and Methods

### Microbial strains

A list of *S. cerevisiae* strains used in this work is given in Table 4. The origin of strains BY4741, BY4742, BY4743, BY4743-20811, BY4743-27248, BY4743-22461, BY4741-811 and BY4741-2461 is the Yeast Knockout Clones and Collection of the Dharmacon^TM^, and were provided by Carlo Erba s.r.l. (Italy). The yeast knock-out mutants were obtained by using a PCR based strategy to replace each ORF with a *kanMX* cassette^48^. The yeast strains were propagated and verified following the manufacturer’s instruction. When required G418 (200 μg/ml) was for strain selection.

*E. coli* JM109 competent cells (Sigma-Aldrich) were used for transformation experiments.

### Media for microbial growth

Yeast strains were cultivated in the following media:

- Yeast-Extract Dextrose-medium (YED): yeast extract 1% [w/v], glucose (dextrose) 2% [w/v].
- Glycerol-complete-medium (YEG): yeast extract 1% [w/v], glycerol 3% [w/v].
- Minimal medium (YNB): yeast Difco nitrogen base without amino acids 0.67% [w/v], glucose 2% [w/v]. Supplements were added when required.

To obtain solid media, 2.5% [w/v] Bacto agar was used.

Bacterial strains were grown in L-broth as previously described by Del Giudice^49,50^.

### Tellurium precipitation test

Stock solutions of K_2_TeO_3_ were prepared, filter sterilized and stored as described^15^.

The tellurium precipitation assays were carried out with sterile microtiter plates (Falcon). In each microtiter wells 0.2 ml of YED broth containing about 2 × 10^5^ yeast cells and increasing amounts of K_2_TeO_3_ were placed. Microtiter plate cultures were incubated at 28 °C without shaking, and Te^0^ precipitation was analyzed after 5-6 days.

### Electron microscopy

TEM Images were obtained as described^15^.

### N-methyl-N’-nitro-N-nitrosoguanidine treatment and isolation of Te^R^ mutants

*N*-methyl-*N*′-nitro*-N*-nitrosoguanidine (NTG) mutagenesis was performed according to^51^. After mutagenesis about 10^8^ cells were plated onto YEG medium supplemented with 100 μg/ml K_2_TeO_3_ and incubated at 28 °C. Mutants appearing as papillae above background growth were isolated and purified three times. Growth measurements were carried out by using a Klett-Summerson colorimeter.

### Genetic techniques

Standard genetic techniques were used as described by^52^.

### Nucleic acids

Total RNA was purified from yeast cells with the use of the MagneSil total RNA mini-Isolation System (Promega). Single preparations were performed on 50 mg of frozen starting material, which was grinded to a fine powder under liquid nitrogen before the extraction.

DNA for PCR analysis was purified from *S. cerevisiae* yeast cultures according to^53^, while the method described by^54^ was followed for DNA extraction from *E. coli* cultures.

### DNA manipulations

General DNA manipulations were carried out by earlier published methods^54^. In order to verify the yeast frataxin sequence present in the Sc57-Te_5_^R^ mutant, gene sequences were amplified from DNA extracted from both wild type and mutant *S. cerevisiae* strains, using two PCR primers situated upstream (fra5: 5′-CGAGAAGATAGAGTGTAGC-3′) and downstream of the frataxin coding sequence (fra3: 5′-GATTGGATGCGTTACAAGTG-3′), respectively. Amplification products were gel purified, ligated into pBluescript vector, and transformed into JM109 competent cells following standard procedures. DNA from 10 recombinant clones carrying the wild type derived frataxin gene and 10 recombinants carrying the mutant-derived frataxin sequence were, subsequently, subjected to sequence analysis using universal forward and reverse sequence primers. Alignment of the obtained sequence was performed with ClustalV software.

### RNA microarray analysis

To measure fluctuations in gene expression, Affymetrix genechips (GeneChip Yeast Genome 2.0 Array) were utilized. These Gene Chips provide comprehensive coverage of both *S. cerevisiae* (5,841 probe sets) and *Schizosaccharomyces pombe* (5,021 probe sets). Total RNA extracted from Sc57-Te_5_^R^ strain treated with K_2_TeO_3_ and from an untreated control was used to prepare molecular probes. Three independent RNA extractions were performed on each single yeast sample. Probe preparation and microarray hybridization were performed in the laboratory of the University of Bristol, following manufacturer’s instructions. Microarray data were analyzed using the FlexArray 1.6.1 software suite (Génome Québec and McGill University). Raw data were normalized using the PLIER (Probe Logarithmic Intensity Error) algorithm. This algorithm is the latest in a series of algorithms produced by Affymetrix to analyze their oligonucleotide GeneChips and employs M-estimators to obtain estimates of probe affinity and target signal. Significant expression differences were identified by means of t-tests. Only differently expressed sequences with p-values < 0.01 and expression ratios >2.0 were taken into consideration. Differential gene expression was assessed using «The Database for Annotation, Visualization and Integrated Discovery (DAVID) v6.8» (https://david.ncifcrf.gov/), in order to enrich the data obtained and deduce groups of genes with altered expression values due to the applied treatment.

### Whole-genome sequencing

Whole-genome sequences of *S. cerevisiae* strains Sc57 and in Sc57-Te_5_^R^ were determined by Next Generation Sequencing (NGS). Preparation of indexed libraries and whole-genome shotgun sequencing were performed as a service by Genomix4Life S.r.l. using an Illumina platform. Samples were assessed after dilution by using Nanodrop 2000c spectrophotometer and Agilent 4200 TapeStation. After sequencing and quality check control of raw sequence data, paired-end reads were aligned against reference genome of strain *S. cerevisiae* S288C^23^ to identify single nucleotide variants (SNVs) and small insertions and deletions (InDels) using gold standard procedures. All SNVs abd InDels are listed in Supplementary Datasets S1 and S2. Alignments were performed with BWA^55^, and SAMtools^56^ and BEDtools^57^ were used for filtering steps and file formats conversion. Reference genome of strain *S. cerevisiae* S288C (assembly R64) was used for functional annotation. Mapping of SNVs and InDels on *S. cerevisiae* coding sequences (CDSs) and intergenic genome regions, and classification of variants based on their effects on CDSs were obtained by using *ad hoc* developed Python programs.

### Data availability

All data generated or analysed during this study are included in this published article (and its Supplementary Information files).

## Electronic supplementary material

Supplementary Figures

Supplementary Dataset S1 (Sc57)

Supplementary Dataset S2 (Sc57-Te5R)

Supplementary Table S1 (up-regulated genes)

Supplementary Table S2 (down-regulated genes)

Supplementary Table S3 (mutations distinguishing SC57Te5R from SC57)
