# Supplementary material for: Mitochondrial ribosomal proteins involved in tellurite resistance in yeast Saccharomyces cerevisiae
Source: Sci Rep. 2018 Aug 13;8:12022. doi: 10.1038/s41598-018-30479-6 (PMC6089990; doi:10.1038/s41598-018-30479-6)
Supplement: Supplementary file 1 — Supplementary Figures [file 41598_2018_30479_MOESM1_ESM.pdf]

Mitochondrial ribosomal proteins involved in tellurite resistance in yeast *Saccharomyces cerevisiae*

Paola Pontieri[1], Hans Hartings[2], Marco Di Salvo[3], Domenica R. Massardo[1], Mario De Stefano[4], Graziano Pizzolante[5], Roberta Romano[6], Jacopo Troisi[7], Angelica Del Giudice[8], Pietro Alifano[3], Luigi Del Giudice[1]*

[1]Istituto di Bioscienze e BioRisorse-UOS Portici-CNR c/o Dipartimento di Biologia, Sezione di Igiene, Via Mezzocannone 16, Napoli 80134, Italy; [2]Consiglio per la ricerca in agricoltura e l'analisi dell'economia agraria, Via Stezzano 24, Bergamo 24126, Italy; [3]Dipartimento di Scienze e Tecnologie Biologiche e Ambientali, Università del Salento, Lecce 73100, Italy; [4]Department of Environmental Sciences, Second University of Naples, Via A. Vivaldi 43, Caserta 81100, Italy; [5]ZooPlantLab, Department of Biotechnology and Biosciences, University of Milano-Bicocca, Piazza della Scienza 2, Milano 20126, Italy; [6]Department of Civil, Chemical, Environmental and Materials Engineering (DICAM), University of Bologna, Via Terracini 28, Bologna 40131, Italy; [7]Theoreo srl – Spin off of the University of Salerno, Via Salvatore Derenzi 50, Montecorvino Pugliano 84125 (SA) Italy; [8]Amb di allergologia Osp Martini asl città di Torino, via Tofane 71,Torino 10171, Italy.

Email: Paola Pontieri: paola.pontieri@ibbr.cnr.it; Hans Hartings: hans.hartings@entecra.it; Marco Di Salvo: marco.disalvo@unisalento.it; Domenica Rita Massardo: dr.massardo@libero.it; Mario De Stefano: mario.destefano@unina2.it; Graziano Pizzolante: gpizzolante79@gmail.com; Roberta Romano: robertaro@libero.it; Jacopo Troisi: jacopo132@gmail.com; Angelica Del Giudice: angelica_delgiudice@libero.it; Pietro Alifano: pietro.alifano@unisalento.it; Luigi Del Giudice*: luigi.delgiudice@ibbr.cnr.it

*** Corresponding author**

Supplementary information file

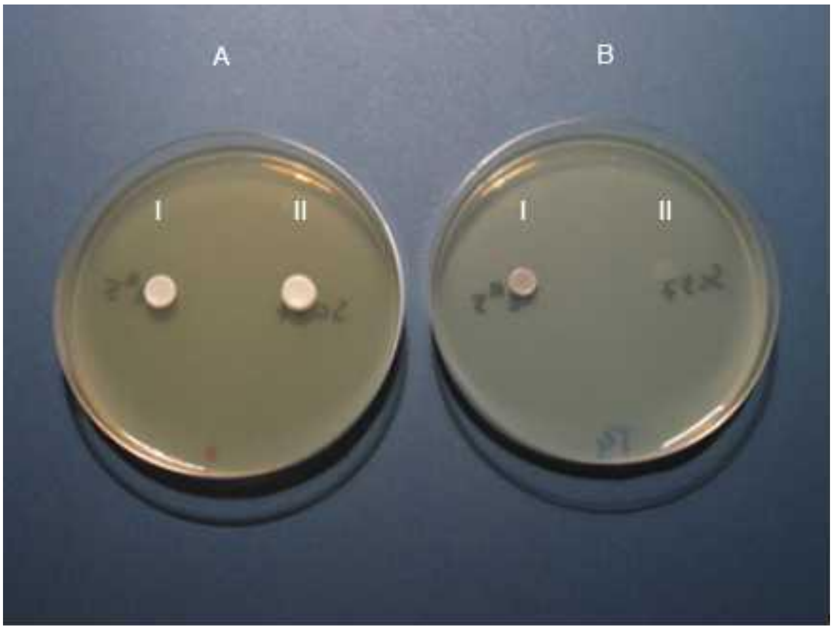

Figure S1. Dropping test of resistance to tellurite of *Saccharomyces cerevisiae* strains. Plate A (YEG agar without tellurite), Plate B (YEG agar plus tellurite). Sc57-Te_5^R strain (I) and Sc57-Te^S strain (II).

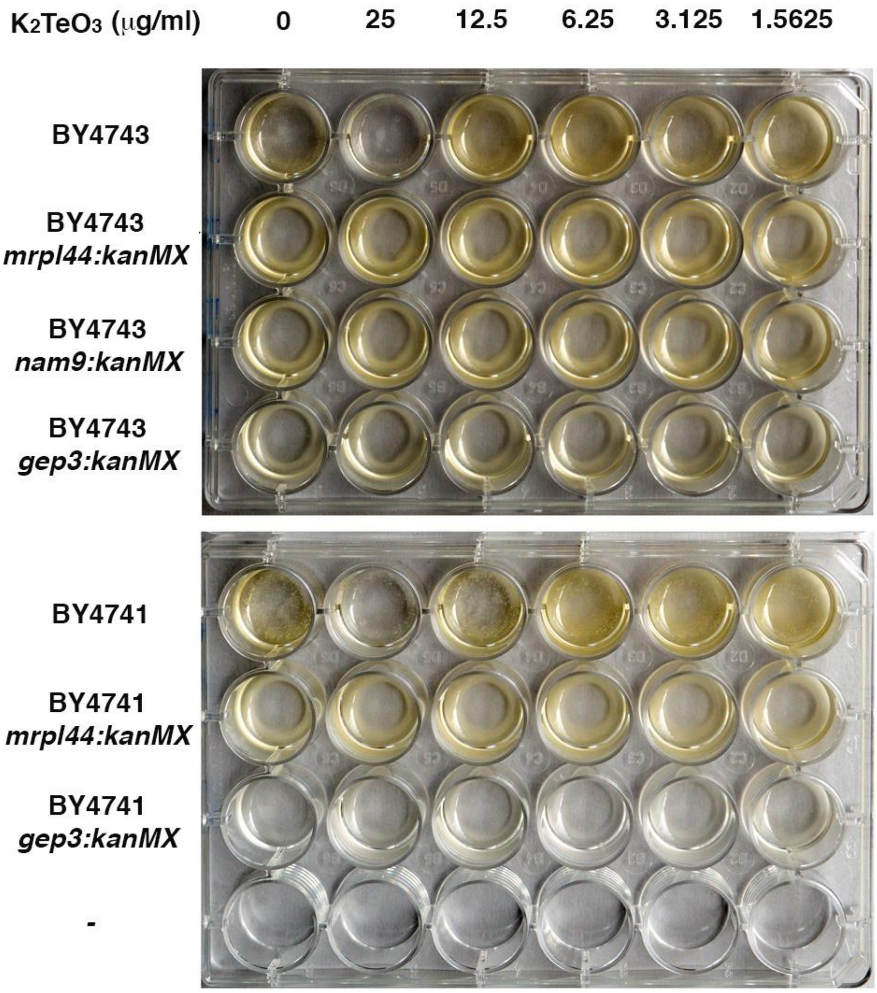

Figure S2. Tellurite resistance test in microtiter plates. Indicated Saccharomyces cerevisiae strains were grown in non-fermentable YEG broth either in the absence or in the presence of increasing amounts of K2TeO3.
